# ﻿*Oreonectesdamingshanensis* (Cypriniformes, Nemacheilidae), a new species of stream fish from Guangxi, Southwest China

**DOI:** 10.3897/zookeys.1180.104645

**Published:** 2023-09-18

**Authors:** Jing Yu, Tao Luo, Chang-Ting Lan, Jia-Jun Zhou, Huai-Qing Deng, Ning Xiao, Jiang Zhou

**Affiliations:** 1 School of Life Sciences, Guizhou Normal University, Guiyang 550025, Guizhou, China; 2 School of Karst Science, Guizhou Normal University, Guiyang 550001, Guizhou, China; 3 Zhejiang Forest Resource Monitoring Center, Hangzhou 310020, Zhejiang, China; 4 Zhejiang Forestry Survey Planning and Design Company Limited, Hangzhou 310020, Zhejiang, China; 5 Guiyang Healthcare Vocational University, Guiyang 550081, Guizhou, China

**Keywords:** Morphology, new species, *Oreonectesplatycephalus* complex, phylogeny, taxonomy

## Abstract

In this work, a new species of the genus *Oreonectes* is described, named *Oreonectesdamingshanensis* Yu, Luo, Lan, Xiao & Zhou, **sp. nov.**, collected from the Damingshan Mountains of the Guangxi Zhuang Autonomous Region, China. Phylogenetic trees constructed based on the mitochondrial Cyt *b* showed that the new species represents an independent evolutionary lineage, with uncorrected genetic distances (*p*-distance) from congeners ranging from 6.1% to 8.9%. Morphologically, the new species can be distinguished from five other species of the genus by a combination of characters. The discovery of this new species raises the number of known species of *Oreonectes* from five to six. Our study suggests that *O.platycephalus* may be a complex containing multiple species and that previously recorded areas need to be further delimited and reevaluated.

## ﻿Introduction

A globally important biodiversity hotspot, the karst region of southwest China is characterized by extremely high species diversity and endemism (Wang et al. 2018). Rivers and mountains as geographic isolation are potential environmental drivers of species formation and diversification (Antonelli et al. 2018), which is also reflected in freshwater fishes from the karsts of southwest China (Wen et al. 2022). Thus, the unique geomorphological and hydrological conditions of karst may have led to the isolation of different geographical populations of widely distributed species, resulting in speciation (Jiang et al. 2022; Li et al. 2022). The large number of new fish species found in the karst region of southwest China over the past decade (Lan et al. 2013; Zhang et al. 2019) also suggests that the species diversity of its freshwater fishes may have been underestimated. Therefore, researchers are required to carry out detailed field surveys for taxonomic studies to assess the potential biodiversity of the area.

**Figure A1.**
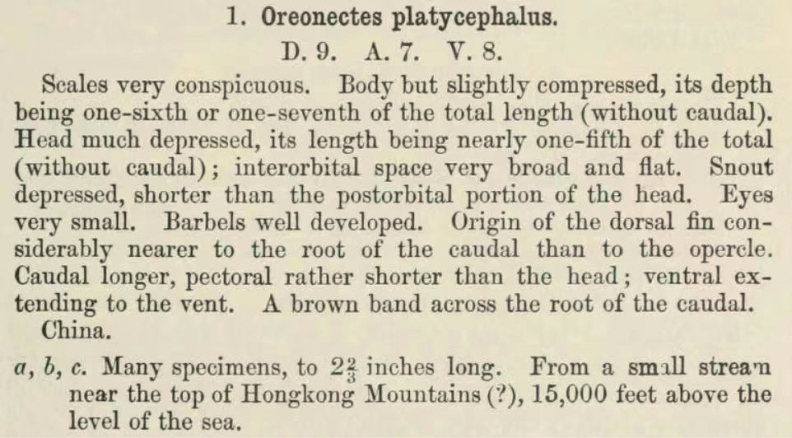
The original description of the *Oreonectesplatycephalus* Zhu, 1989. Provided by J-J Zhou, February 13, 2023.

The small loaches of the genus *Oreonectes* Günther, 1868 (Cypriniformes, Nemacheilidae), typically have a total length of ~80 mm (Zhu 1989; Lan et al. 2013). The genus *Oreonectes* Günther, 1868 was initially described based on specimens collected from Hong Kong, with the type species *Oreonectesplatycephalus* Günther, 1868 (Günther 1868) (Appendix 2: Fig. A1). However, the classification of the genus *Oreonectes* remains controversial and unresolved. Recently, Luo et al. (2023) conducted a phylogenetic study, which included a large genetic sample, based on mitogenome and seven nuclear genes, that largely clarified the phylogenetic relationships between species within the genera *Oreonectes* and *Troglonectes* Zhang, Zhao & Tang, 2016. Currently, there are five recognized species in the genus *Oreonectes* distributed in mountain streams in Guangxi, Guangdong, and Hong Kong in China, and in northern Vietnam (Zhu 1989; Lan et al. 2013), namely *O.guananensis* Yang, Wei, Lan & Yang, 2011, *O.guilinensis* Huang, Yang, Wu & Zhao, 2020, *O.luochengensis* Yang, Wu, Wei & Yang, 2011, *O.platycephalus* Günther, 1868, and *O.polystigmus* Du, Chen & Yang, 2008 (Du et al. 2008; Günther 1868; Huang et al. 2020; Zheng 1981; Zhu 1989; Yang et al. 2011a, b). The genus *Troglonectes*, after revision by from Luo et al. (2023), includes only 16 species, *T.barbatus*, *T.canlinensis*, *T.daqikongensis*, *T.elongatus*, *T.furcocaudalis*, *T.hechiensis*, *T.huanjiangensis*, *T.lihuensis*, *T.lingyunensis*, *T.longibarbatus*, *T.macrolepis*, *T.maolanensis*, *T.microphthalmus*, *T.retrodorsalis*, *T.shuilongensis*, and *T.translucens* (Li et al. 2023).

**Figure 1.**
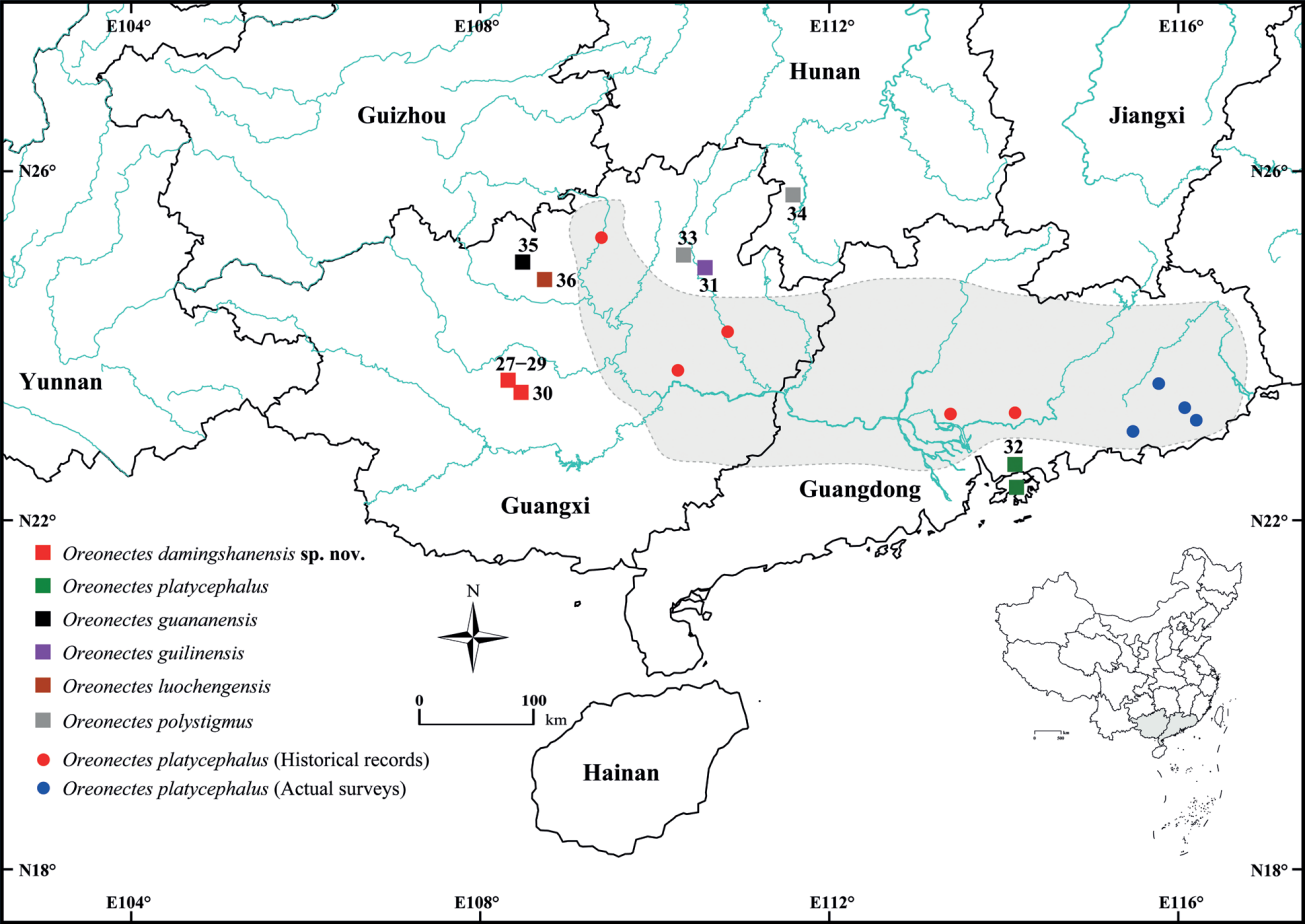
Sampling collection localities and distribution of *Oreonectesdamingshanensis* sp. nov. and five species of the genus *Oreonectes* in southern China. For details of ID numbers, please see Table 2. The green rectangular box without an ID is the type locality of *O.platycephalus*. The base maps are from the Standard Map Service website (http://bzdt.ch.mnr.gov.cn/index.html).

Between 2020 and 2023, we collected 30 specimens during field surveys of open streams in Nanning City, Guangxi Zhuang Autonomous Region, China (Fig. 1). Through examination of these specimens and molecular sequencing, we observed significant differences in both morphology and mitochondrial cytochrome b gene (Cyt *b*) in these specimens when compared with their congeners. These differences led us to identify this as a new species of the genus *Oreonectes*, which we named *Oreonectesdamingshanensis* sp. nov.

## ﻿Materials and methods

### ﻿DNA extraction, PCR, and sequencing

Genomic DNA was extracted from muscle tissue using a DNA extraction kit from Tiangen Biotech (Beijing) Co. Ltd. Two muscle samples of the new species were sequenced for the mitochondrial cytochrome b gene (Cyt *b*). The forward and reverse primers used for Cyt *b* were F14724 (5’-GACTTGAAAAACCACCGTTG-3’) and R15915 (5’-CTCCGATCTCCGGATTACAAGAC-3’), respectively, following Xiao et al. (2001). PCR amplifications were performed in a 25 μl reaction volume with the following cycling conditions: an initial denaturing step at 95 °C for five min, 36 cycles of denaturing at 95 °C for 40 s, annealing at 45 °C for 40 s and extending at 72 °C for 1 min, and a final extension at 72 °C for 10 min. The purified products were sequenced with both forward and reverse primers using a BigDye Terminator Cycle Sequencing Kit according to the manufacturer’s instructions. The products were sequenced on an ABI Prism 3730 automated DNA sequencer by Chengdu TSING KE Biological Technology Co. Ltd. (Chengdu, China). All sequences have been deposited in GenBank (Table 2).

**Table 1.** Species list of the genus *Oreonectes* and comparisons of diagnostic characters of the new species with congeners. Grey shading indicates a clear difference in a character compared to that of *Oreonectesdamingshanensis* sp. nov.

Species	Body pigmentation	Eyes	Scales	Dorsal-fin rays	Pectoral-fin rays	Pelvic-fin rays	Anal-fin rays	Caudal-fin rays	Caudal fin	Caudal fin with irregular black markings
*Oreonectesdamingshanensis* sp. nov.	Present	Normal	Present	iii, 7	i, 9	i, 7	iii, 5	14	Rounded	Yes
*O.guananensis* Yang, Wei, Lan & Yang, 2011	Present	Normal	Present	iii, 7	i, 10–11	i, 7–8	iii, 5	13–17	Rounded	No
*O.guilinensis* Huang, Yang, Wu & Zhao, 2020	Present	Normal	Present	ii, 6	i, 9–10	i, 6	iii, 5	13–14	Rounded	Yes
*O.luochengensis* Yang, Wu, Wei & Yang, 2011	Absent	Normal	Present	iii, 7	i, 11–12	i, 7	ii, 5	14–16	Truncated	No
*O.platycephalus* Günther, 1868	Present	Normal	Present	iii, 8–9	i, 11	i, 8	ii, 6–7	13–15	Rounded	No
*O.polystigmus* Du, Chen & Yang, 2008	Present	Normal	Present	iii, 6–7	i, 10	i, 6	ii, 5	14–15	Rounded	No
	**Tip of pelvic fin reaching anus**		**Dorsal fin origin**			**Maxillary barbel**			**Reference**	
*Oreonectesdamingshanensis* sp. nov.	No		Posterior to the pelvic-fin origin			Not reaching to posterior margin of gill cover			This study	
*O.guananensis* Yang, Wei, Lan & Yang, 2011	No		Opposite to pelvic-fin origin			Reaching to the gill cover			Yang et al. 2011a; This study	
*O.guilinensis* Huang, Yang, Wu & Zhao, 2020	Yes		Slightly posterior to the pelvic-fin origin			Reaching to the posterior margin of the eye			Huang et al. 20120	
*O.luochengensis* Yang, Wu, Wei & Yang, 2011	No		Slightly posterior to the pelvic-fin origin			Reaching to the posterior margin of the eye			Yang et al. 2011b; This study	
*O.platycephalus* Günther, 1868	No		Posterior to the pelvic-fin origin			Reaching to the posterior margin of the eye			Günther 1868; This study	
*O.polystigmus* Du, Chen & Yang, 2008	Yes		Slightly posterior to the pelvic-fin origin			Reaching to the pectoral-fin origin			Du et al. 2008; This study	

**Table 2.** Localities, voucher information, and GenBank numbers for all samples used.

ID	Genus	Species	Localities (* type localities)	Voucher	GenBank
1	*Troglonectes*	*Troglonecteselongatus*	Mulun Town, Huanjiang County, Guangxi, China*	GZNU 2020073101	ON116502
2		*Troglonecteslihuensis*	Lihu Town, Nandan County, Guangxi, China*	GZNU 2019011211	ON148332
3		*Troglonectesdaqikongensis*	Chaoyang Town, Libo County, Guizhou, China*	GZNU 2019011207	ON116526
4		*Troglonecteshechiensis*	Tongjin Village, Hechi City, Guangxi, China*	D1820	MW495267
5		*Troglonectesretrodorsalis*	Liuzhai Town, Nandan County, Guangxi, China*	GZNU 2020073103	ON116511
6		*Troglonectesshuilongensis*	Shuilong Town, Sandu County, Guizhou, China*	GZNU 2019011201	ON116522
7		*Troglonectesmacrolepis*	Dacai Town, Huanjiang County, Guangxi, China*	GZNU 2019122202	ON116498
8		*Troglonectesmicrophthalmus*	Tianhe Town, Luocheng County, Guangxi, China*	GZNU 2020041601	ON116494
9		*Troglonectestranslucens*	Xiaao Town, Duan County, Guangxi, China*	GZNU 2020082302	ON116510
10		*Troglonectesbarbatus*	Lihu Town, Nandan County, Guangxi, China*	GZNU 2020011503	ON116501
11		*Troglonectesfurcocaudalis*	Yongle Town, Rongshui County, Guangxi, China*	GZNU 2020042701	ON116512
12	*Paranemachilus*	*Paranemachiluspingguoensis*	Changping Town, Fusui County, Guangxi, China*	GZNU 2019122205	ON116500
13		*Paranemachiluszhengbaoshani*	Duan County, Guangxi, China	GZNU20210526001	ON116530
14		*Paranemachilusgenilepis*	Guohua Town, Pingguo County, Guangxi, China*	GZNU 2019122206	ON116497
15	*Yunnanilus* (I)	*Yunnaniluslongidorsalis*	Agang Longtan pool, Luoping County, Yunnan, China	N/A	NC_062728
16		*Yunnanilusniger*	N/A	N/A	NC_063106
17		*Yunnanilusjiuchiensis*	Jiuchi County, Penzhou City, Sichuan, China	N/A	MW532080
18	*Karstsinnectes*	*Karstsinnectesparva*	Ande Town, Jingxi City, Guangxi, China	Tissue ID: JTQ02	ON116520
19		*Karstsinnectesacridorsalis*	Bamu Town, Tiane County, Guangxi, China*	Tissue ID: GZNU2020	ON116515
20		*Karstsinnectesanophthalmus*	Leiping Town, Daxin County, Guangxi, China*	GZNU 2019011310	ON116513
21		*Karstsinnectesanophthalmus*	Leiping Town, Daxin County, Guangxi, China*	GZNU 2019011210	ON148333
22		*Karstsinnectesanophthalmus*	Chengxiang Town, Wuming County, Guangxi, China*	GZNU 2019122201	ON116506
23	*Micronemacheilus*	*Micronemacheiluscruciatus*	N/A	N/A	AP012142
24		*Micronemacheiluspulcherrimus*	Duan County, Hechi City, Guangxi, China	GZNU20210609004	ON116493
25	*Yunnanilus* (II)	*Yunnanilusbailianensis*	Bailian cave, Liuzhou City, Guangxi, China*	GZNU 2020041603	ON116504
26		*Yunnaniluslongibarbatus*	Gaoling Town, Duan County, Guangxi, China*	GZNU 2020073104	ON116508
27	*Oreonectes*	*Oreonectesdamingshanensis* sp. nov.	Leping Village, Guling Town, Mashan County, Guangxi, China*	GZNU20230216010	OQ754116
28		*Oreonectesdamingshanensis* sp. nov.	Leping Village, Guling Town, Mashan County, Guangxi, China*	GZNU20230216011	OQ754117
29		*Oreonectesdamingshanensis* sp. nov.	Leping Village, Guling Town, Mashan County, Guangxi, China*	GZNU20230216012	OQ754118
30		*Oreonectesdamingshanensis* sp. nov.	Damingshan Mountain, Shanglin County, Guangxi, China	GZNU 2020112502	ON116496
31		*Oreonectesguilinensis*	Shigumen Village,Xingping Town,Yangshuo County, Guangxi, China*	N/A	MN239094
32		*Oreonectesplatycephalus*	Shenzhen City, Guangdong, China*	GZNU 2020112501	ON116528
33		*Oreonectespolystigmus*	Dabu Town, Yanshan District, Guilin, Guangxi, China*	GZNU 2020011501	ON116514
34		*Oreonectespolystigmus*	Jianghua County, Yongzhou City, Hunan, China	GZNU20210609005	ON116517
35		*Oreonectesguananensis*	Changmei Town, Huanjiang County, Guangxi, China*	GZNU 2020073102	ON116507
36		*Oreonectesluochengensis*	Tianhe Town, Luocheng County, Guangxi, China*	GZNU 2020011502	ON116495
37		*Lefuacostata*	N/A	N/A	KT943751
38		*Lefuanikkonis*	N/A	CBM: ZF 11290	AP011300
39		*Lefuaechigonia*	Hino, Shiga, Japan	N/A	AB054126
40	Outgroup	*Traccatichthyspulcher*	Leiping Town, Daxin County, Guangxi, China	Tissue ID: GX1	ON116516
41		*Triplophysabaotianensis*	Nanpanjiang River, Panzhou City, Guzihou, China*	GZNU20180421005	MT992550

### ﻿Phylogenetic analyses

A total of 41 Cyt *b* sequences were used for phylogenetic analysis. In addition to the four new sequences, the remaining 37 sequences were downloaded from GenBank and included five already recognized genera (Table 2) and two outgroup species from the mitogenome provided by Luo et al. (2023).

Mitochondrial Cytb sequences were aligned in MEGA v7.0 (Kumar et al. 2016) by the MUSCLE (Edgar 2004) algorithm with default parameters. Phylogenetic trees were constructed using both maximum likelihood (ML) and Bayesian inference (BI) methods. The ML tree was conducted in IQ-TREE v2.0.4 (Nguyen et al. 2015) with 2000 ultrafast bootstrap (UFB) replicates (Hoang et al. 2018) and was run until a correlation coefficient of at least 0.99 was reached. The BI phylogeny was constructed in MrBayes v3.2.1 (Ronquist et al. 2012). Two independent runs were conducted in the BI analysis, each of which was performed for 2 × 10^7^ generations and sampled every 1000 generations. The first 25% of the samples was discarded as a burn-in, resulting in a potential scale reduction factor of < 0.01. For BI and ML analyses, the best-fit model was obtained based on the Bayesian information criterion computed with PartitionFinder v2.1.1 (Lanfear et al. 2017). In this analysis, the first, second, and third codons of the Cyt *b* gene were defined.

The results of the model selection suggested that the first, second, and third codons of the best-fit model for the Cyt *b* gene were K80+I+G, HKY+I+G, and TRN+I+G, respectively. Nodes in the trees were considered well supported when Bayesian posterior probabilities (BPP) were ≥ 0.95 and the ML ultrafast bootstrap value (UBP) was ≥ 9 5%. Uncorrected *p*-distances (1000 replicates) based on the Cyt *b* gene were calculated using MEGA 7.0 (Kumar et al. 2016).

### ﻿Morphological comparisons

Morphometric data were collected from 53 well-preserved specimens of the genus *Oreonectes* (Appendix 3: Table A1). A total of 33 measurements were recorded to the nearest 0.1 mm with digital calipers following the protocol of Tang et al. (2012). All measurements were taken on the left side of the fish specimens.

Comparative data for the five species of the genus *Oreonectes* were obtained from the literature and specimen examination (Table 4). Specimens of four species from the type locality were examined, including *O.guananensis*, *O.luochengensis*, *O.platycephalus*, and *O.polystigmus* (see Appendix 1). Considering the morphological similarity of the new species to *O.platycephalus* and *O.polystigmus*, the measurements were also included in the statistical analysis. Principal component analyses (PCAs) of size-corrected measurements and simple bivariate scatterplots were used to explore and characterize the morphometric differences between the new species and *O.platycephalus* and *O.polystigmus*. Mann–Whitney *U* tests were used to determine the significance of differences in morphometric characters between the new species and the above two similar species. All statistical analyses were performed using SPSS 21.0 (SPSS, Inc., Chicago, IL, USA), and differences were considered statistically significant at a *p*-value < 0.05. PCAs of morphological data were performed after logarithmic transformation and under conditions of no rotation. In addition, as reported by other researchers (Parsons and Jones 2000; Polaszek et al. 2010), canonical discriminant analysis (CDA, George and Paul 2010) was used to classify individuals into different groups, where a priori membership was determined based on specimens belonging to different species. All pre-processing of morphological data was performed in Microsoft Excel (Microsoft Corporation 2016).

### ﻿X-ray scanning and three-dimensional image reconstructions

In order to obtain information on the skeletons of the new species, X-ray scanning was conducted via nano-computerized tomography. Specimens were scanned using a GE v|tome|x m dual tube 300/180 kv system at the Key Laboratory of Vertebrate Evolution and Human Origins, Institute of Vertebrate Paleontology and Paleoanthropology (IVPP), Chinese Academy of Sciences. Each specimen was scanned with an energy beam of 80 kV and a flux of 80 μA using a 360° rotation, and the data were then reconstructed into a 4096 × 4096 matrix of 1536 slices. The final CT reconstructed skull images were exported with a minimum resolution of 6.099 μm. The skull images were exported from the virtual 3D model and reconstructed by Volume Graphics Studio 3.0.

## ﻿Results

### ﻿Phylogenetic analyses and genetic divergence

**Figure 2.**
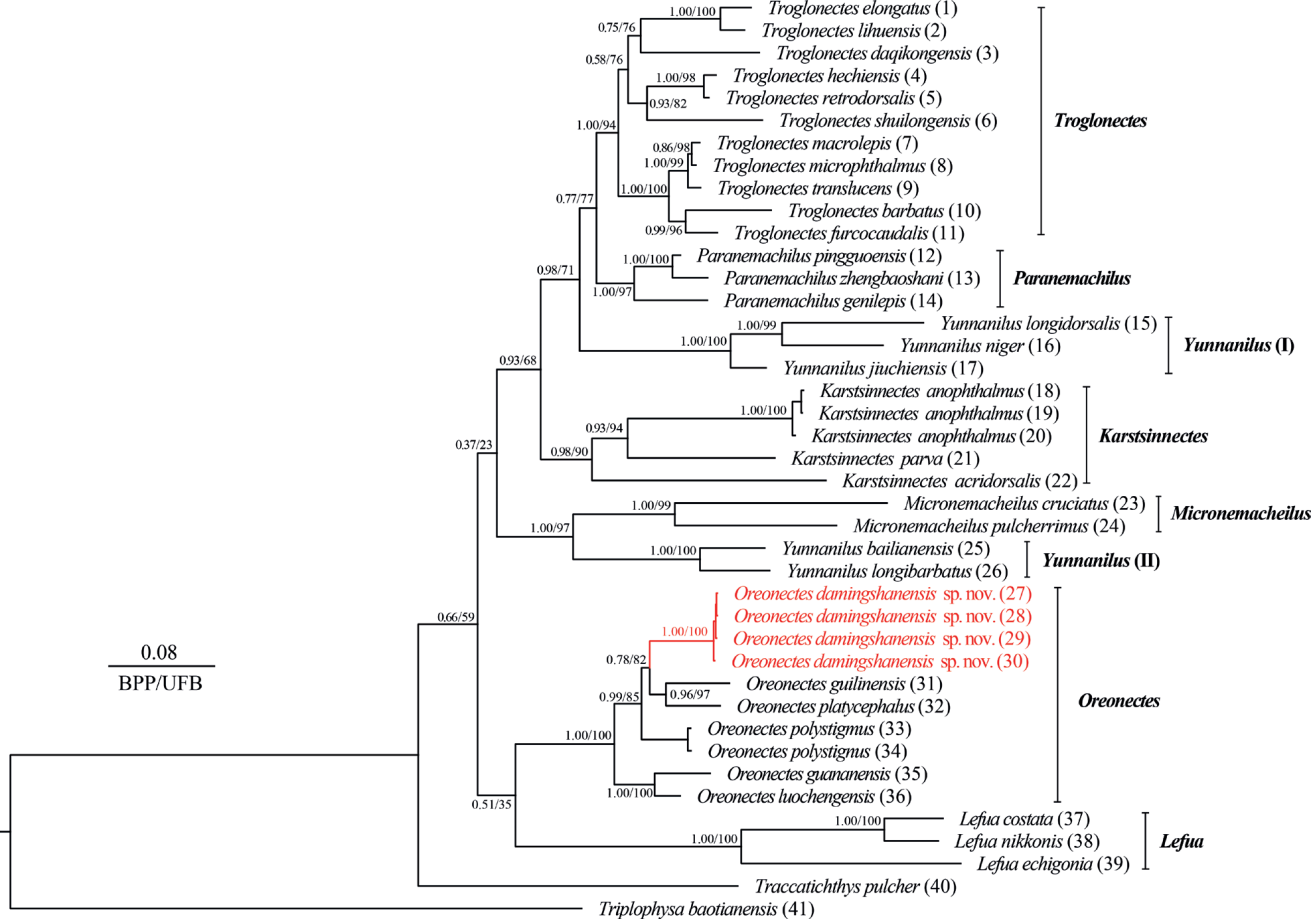
Phylogenetic tree based on mitochondrial Cyt *b* (1140 bp). In this phylogenetic tree, Bayesian posterior probabilities (BPP) from BI analysis/ultrafast bootstrap supports (UFB) from ML analysis are listed beside nodes. The scale bar represents 0.08 nucleotide substitutions per site. The numbers at the tip of branches correspond to the ID numbers listed in Table 2.

BI and ML analyses were performed to construct phylogenetic trees with consistent topologies based on mitochondrial Cyt *b* sequences with a length of 1140 base pairs (Fig. 2). These phylogenetic trees showed a topology similar to that of Luo et al. (2023), but with lower node support between major clades (Fig. 2). In addition, several new clades were identified, and *Yunnanilus* was divided into two distant clades named *Yunnanilus* (I) and *Yunnanilus* (II). *K.anophthalmus*, *K.parva*, and *K.acridorsalis* were clustered together to form a separate clade. In fact, all five remaining species of the genus *Oreonectes*, clustered together to form a sister clade of the genus *Lefua*. Within the genus *Oreonectes*, the four specimens collected from Mashan and Shanglin counties in Nanning City, Guangxi, China, formed a distinct and highly supported clade with *O.platycephalus* and *O.guilinensis* (0.99 in BI and 85% in ML) (Fig. 2).

Within the genus *Oreonectes*, the genetic distances between the new species *Oreonectesdamingshanensis* sp. nov. and the remaining five species range from 6.1% (for *O.polystigmus*) to 8.9% (for *O.guananensis*). This level of divergence was similar to those between pairs of other recognized species. For example, the Cyt *b p*-distance was 4.9% between *O.luochengensis* and *O.guananensis* (Table 3).

**Table 3.** Uncorrected *p*-distances (%) between five species of the genus *Oreonectes* based on the mitochondrial Cyt *b* gene.

ID	Species	1	2	3	4	5
1	*Oreonectesdamingshanensis* sp. nov.					
2	*Oreonectesguananensis*	8.9				
3	*Oreonectesguilinensis*	7.2	8.8			
4	*Oreonectesluochengensis*	7.7	4.9	8.1		
5	*Oreonectespolystigmus*	6.1	8.6	7.4	7.6	
6	*Oreonectesplatycephalus*	6.8	8.8	6.5	8.0	6.5

### ﻿Morphological analyses

**Figure 3.**
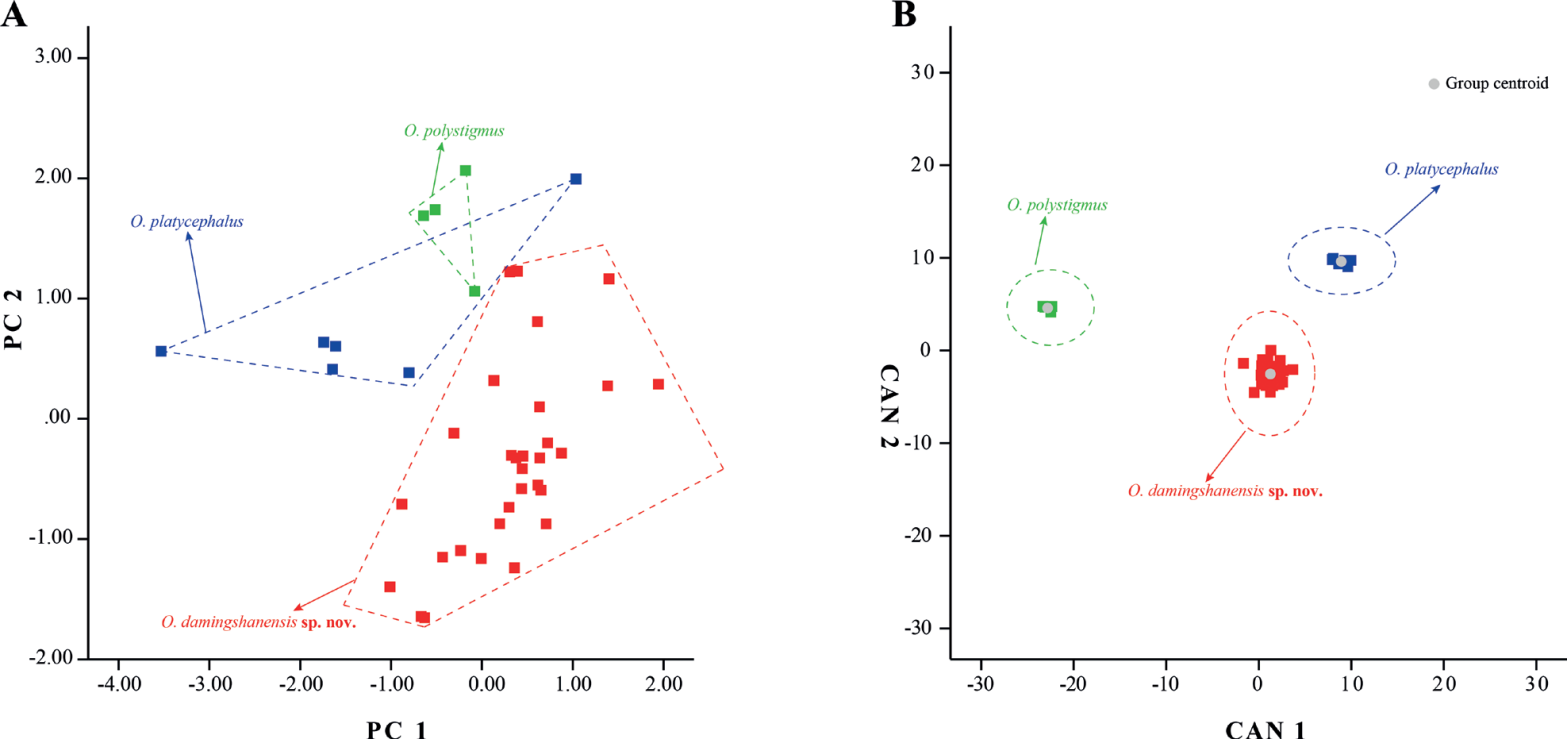
Plots from the principal component analysis, and canonical discriminant analysis scores of *Oreonectesdamingshanensis* sp. nov., *O.polystigmus*, and *O.platycephalus* based on morphological characters.

Mann–Whitney *U* tests showed that the *Oreonectesdamingshanensis* sp. nov. differed from *O.luochengensis*, *O.polystigmus*, *O.guananensis*, and *O.platycephalus* in several morphological characters (Table 4). These significant differences were mainly observed in the head, fins, and tail regions. The differences were more pronounced in comparisons of the new species with *O.platycephalus*, with 84.8% of the morphological characters being significantly different (*p* = 0.00−0.046) (Table 4). Based on PCA of the morphological data, two principal component factors with eigenvalues greater than one were extracted. These accounted for 84.09% of the total variation (Appendix 4: Table A2). The first principal component (PC1) accounted for 77.92% of the variation and was positively correlated with all variables (eigenvalue = 28.91), thus reflecting the morphological differences between *Oreonectesdamingshanensis* sp. nov. and similar species. This axis corresponded to body length, head, fins, nostrils, and barbel length. Thus, based on the statistical analysis of the measurements and the PCA and CDA results, 30 specimens from *Oreonectesdamingshanensis* sp. nov. were clearly distinguished via morphological characters from the four similar species *O.luochengensis*, *O.polystigmus*, *O.guananensis*, and *O.platycephalus*. The second principal component (PC2) accounted for 6.17% of the variation and was influenced by the length of the distance between posterior nostrils, length of the upper jaw, mouth width, and eye width (eigenvalue = 0.04) (Appendix 4: Table A2). The two-dimensional plots of PC1 and PC2 clearly separated *Oreonectesdamingshanensis* sp. nov. from *O.polystigmus* and *O.platycephalus* (Fig. 3A). CDA correctly classified 100% of the individuals in the initial grouping case for the three sample groups (*N* = 40). Canonical axes (CAN) 1–2 explained 75.8% and 24.2% of the total variation (Fig. 3B; Appendix 4: Table A2).

**Table 4.** Morphological comparisons of *Oreonectesdamingshanensis* sp. nov. (*OD*), *O.luochengensis* (*OL*), *O.guananensis* (*OG*), *O.platycephalus* (*OPL*), and *O.polystigmus* (*OPO*). All units in mm. *P*-values are at 95% significance.

	*O.damingshanensis* (*OD*)		*O.luochengensis* (*OL*)		*O.guananensis* (*OG*)		*O.platycephalus* (*OPL*)		*O.polystigmus* (*OPO*)		*P*-value from Mann-Whitney U test			
	Range	Mean ± SD	Range	Mean ± SD	Range	Mean ± SD	Range	Mean ± SD	Range	Mean ± SD	*OD vs OL*	*OD vs OG*	*OD vs OPL*	*OD vs OPO*
Total length	55.7–98.9	73.5 ± 9.5	61.7–80.6	72.1 ± 8.2	50.5–89.0	68.4 ± 18.1	41.0–85.5	56.3 ± 15.1	61.5–66.9	65.3 ± 2.5	0.909	0.536	0.005	0.073
Standard length	46.2–81.8	60.6 ± 8.1	51.7–66.2	59.5 ± 6.2	40.6–73.8	56.7 ± 14.8	33.1–73.6	48.1 ± 13.8	51.2–55.5	54.2 ± 2.0	0.802	0.567	0.012	0.093
Body depth	7.0–14.8	9.4 ± 1.7	6.2–9.3	8.1 ± 1.3	4.5–9.8	7.4 ± 2.2	4.6–12.2	7.0 ± 2.6	6.0–11.4	8.8 ± 2.3	0.128	0.086	0.006	0.699
Body width	4.6–10.2	6.7 ± 1.2	6.3–9.4	7.2 ± 1.3	4.2–6.9	5.6 ± 1.2	2.8–7.6	4.5 ± 1.7	5.5–8.7	7.0 ± 1.4	0.802	0.069	0.005	0.738
Head length	10.2–16.6	13.3 ± 1.7	10.6–13.2	12.1 ± 1.0	5.1–16.8	11.7 ± 4.9	6.6–16.7	10.1 ± 3.5	10.2–12.5	11.6 ± 1.0	0.069	0.536	0.010	0.022
Head depth	5.4–9.5	7.2 ± 1.0	5.6–7.3	6.5 ± 0.6	3.3–6.5	4.7 ± 1.5	2.9–6.5	4.8 ± 1.3	5.7–7.8	6.8 ± 1.0	0.069	0.001	0.000	0.485
Head width	7.7–13.4	9.7 ± 1.3	5.6–7.6	6.7 ± 0.9	4.3–8.6	7.0 ± 1.8	4.5–9.1	6.7 ± 1.5	7.4–8.1	7.7 ± 0.3	0.000	0.002	0.000	0.000
Distance between anterior nostrils	2.8–6.5	3.9 ± 0.7	2.3–3.3	2.9 ± 0.4	1.8–4.0	2.9 ± 1.0	1.7–3.8	2.4 ± 0.8	2.4–2.9	2.7 ± 0.2	0.002	0.043	0.001	0.000
Distance between posterior nostrils	3.4–6.5	4.7 ± 0.7	3.1–4.8	3.9 ± 0.7	2.6–6.6	4.5 ± 1.5	2.3–6.1	4.0 ± 1.3	4.2–5.1	4.5 ± 0.4	0.022	0.697	0.052	0.453
Distance between anterior and posterior nostrils	0.6–1.5	0.9 ± 0.2	1.1–1.4	1.2 ± 0.1	1.2–1.8	1.4 ± 0.2	0.6–1.5	0.9 ± 0.3	1.0–1.2	1.1 ± 0.1	0.001	0.000	0.918	0.015
Snout length	4.2–7.1	5.5 ± 0.8	4.1–7.7	5.3 ± 1.4	3.6–6.8	5.1 ± 1.3	1.9–7.0	4.0 ± 1.7	4.4–10.7	6.1 ± 3.1	0.321	0.448	0.006	0.338
Upper jaw length	2.2–4.1	3.2 ± 0.4	2.3–2.9	2.6 ± 0.3	1.7–3.7	2.6 ± 0.8	1.2–3.5	2.0 ± 0.8	2.1–2.6	2.3 ± 0.2	0.004	0.185	0.003	0.001
Lower jaw length	0.3–3.3	2.4 ± 0.5	1.8–2.5	2.2 ± 0.3	1.5–2.8	2.1 ± 0.5	0.9–2.4	1.5 ± 0.5	1.5–1.9	1.7 ± 0.2	0.116	0.155	0.001	0.001
Mouth width	2.5–6.4	4.7 ± 0.8	3.1–4.0	3.7 ± 0.3	2.0–4.4	3.1 ± 1.0	1.6–4.1	2.8 ± 0.8	2.6–3.6	3.1 ± 0.4	0.003	0.003	0.000	0.001
Eye diameter	0.9–2.0	1.6 ± 0.3	5.5–6.6	6.0 ± 0.5	3.7–7.8	5.8 ± 1.9	0.5–1.9	1.1 ± 0.6	0.8–1.1	0.9 ± 0.2	0.000	0.000	0.046	0.001
Interorbital distance	4.4–7.7	5.6 ± 0.9	1.6–2.2	1.9 ± 0.3	1.1–2.1	1.7 ± 0.4	3.7–8.2	5.2 ± 1.6	5.0–6.8	6.1 ± 0.8	0.000	0.000	0.217	0.218
Predorsal length	27.0–48.7	36.5 ± 5.1	29.8–38.2	34.2 ± 3.6	22.5–42.5	32.4 ± 9.1	19.7–43.1	28.8 ± 7.9	29.6–33.2	31.9 ± 1.7	0.345	0.477	0.016	0.064
Dorsal-fin base length	4.9–9.1	6.2 ± 1.0	4.1–6.2	5.2 ± 1.0	2.5–7.3	5.0 ± 1.8	2.1–6.4	3.9 ± 1.4	4.4–5.9	5.0 ± 0.6	0.069	0.141	0.001	0.008
Dorsal-fin length	8.8–14.7	11.4 ± 1.4	9.4–12.3	10.5 ± 1.2	7.2–14.9	10.9 ± 3.1	4.9–9.9	7.6 ± 1.7	9.0–9.7	9.5 ± 0.3	0.202	0.506	0.000	0.002
Pectoral-fin length	8.1–13.2	10.1 ± 1.3	7.9–11.9	10.1 ± 1.6	5.3–11.5	8.7 ± 2.7	5.1–10.8	7.7 ± 1.9	8.8–9.3	9.1 ± 0.2	0.945	0.421	0.006	0.082
Pectoral-fin base length	1.8–3.5	2.4 ± 0.4	1.6–2.8	2.0 ± 0.5	1.1–2.3	1.8 ± 0.5	1.3–2.7	1.9 ± 0.5	1.6–2.7	2.3 ± 0.5	0.048	0.002	0.014	0.485
Prepectoral length	9.8–17.3	13.2 ± 1.7	11.4–15.1	12.9 ± 1.4	9.6–17.2	13.5 ± 3.3	7.0–17.6	10.6 ± 3.7	11.1–12.8	12.0 ± 0.8	0.598	0.909	0.014	0.117
Pelvic-fin length	6.9–12.3	9.6 ± 1.3	6.6–11.4	8.7 ± 1.8	5.5–10.2	8.0 ± 2.1	5.3–11.1	8.0 ± 1.9	7.1–8.0	7.5 ± 0.4	0.237	0.141	0.052	0.005
Pelvic-fin base length	1.5–3.3	2.3 ± 0.4	1.6–2.0	1.7 ± 0.2	0.8–1.9	1.2 ± 0.5	1.2–2.6	1.7 ± 0.5	1.4–1.7	1.6 ± 0.1	0.001	0.000	0.009	0.001
Prepelvic length	23.8–41.4	31.0 ± 4.3	28.5–36.2	32.2 ± 3.3	22.9–40.4	30.9 ± 8.5	16.8–39.3	24.3 ± 7.9	26.1–30.3	28.8 ± 1.9	0.567	0.631	0.012	0.131
Anal-fin length	8.3–13.6	10.4 ± 1.2	7.5–9.9	8.6 ± 1.0	6.7–12.0	9.0 ± 2.5	4.8–8.7	7.5 ± 1.4	7.6–8.6	8.1 ± 0.4	0.003	0.369	0.000	0.000
Anal-fin base length	4.2–7.2	5.2 ± 0.6	2.0–5.7	3.9 ± 1.4	3.4–5.1	4.2 ± 0.8	2.0–5.2	3.6 ± 1.0	3.9–5.0	4.2 ± 0.6	0.025	0.016	0.001	0.007
Preanal length	34.5–61.3	46.0 ± 6.4	30.7–53.6	42.2 ± 8.2	32.7–57.2	44.3 ± 11.3	24.4–56.0	36.3 ± 10.6	38.1–44.7	42.3 ± 2.9	0.345	0.697	0.012	0.131
Caudal peduncle length	6.4–14.6	9.3 ± 1.6	6.8–8.1	7.4 ± 0.5	7.1–9.0	8.1 ± 0.8	5.6–11.1	7.7 ± 1.9	6.5–9.9	8.8 ± 1.5	0.004	0.048	0.052	0.661
Caudal peduncle depth	4.9–9.5	6.6 ± 1.0	4.7–6.4	5.6 ± 0.7	3.4–8.1	5.7 ± 2.1	3.5–7.6	5.2 ± 1.4	4.4–5.9	5.3 ± 0.7	0.014	0.536	0.018	0.013
Maxillary barbel length	4.4–8.1	5.8 ± 0.9	5.1–6.8	5.8 ± 0.9	3.6–8.9	6.4 ± 2.1	1.8–6.2	3.9 ± 1.6	5.2–6.9	5.9 ± 0.7	0.909	0.421	0.003	0.817
Inrostral barbel length	3.5–5.6	4.5 ± 0.6	3.6–4.7	4.0 ± 0.5	2.2–7.0	4.2 ± 1.8	1.5–5.5	3.3 ± 1.3	4.4–6.1	5.0 ± 0.8	0.105	0.369	0.008	0.239
Outrostral barbel length	5.3–9.7	6.9 ± 1.0	4.5–7.4	5.9 ± 1.2	4.2–9.1	6.8 ± 2.1	3.4–8.1	4.9 ± 1.6	6.0–8.1	7.0 ± 0.9	0.105	0.802	0.005	0.817

## ﻿Taxonomic account

### ﻿ Oreonectes damingshanensis

Taxon classificationAnimaliaCypriniformesNemacheilidae

Yu, Luo, Lan, Xiao & Zhou sp. nov.

EE71EBC4-DCEF-565B-84AE-5977FDADF1F5

https://zoobank.org/FAE4223D-632B-4CE6-8990-6EAD4D410233

**Figure 4.**
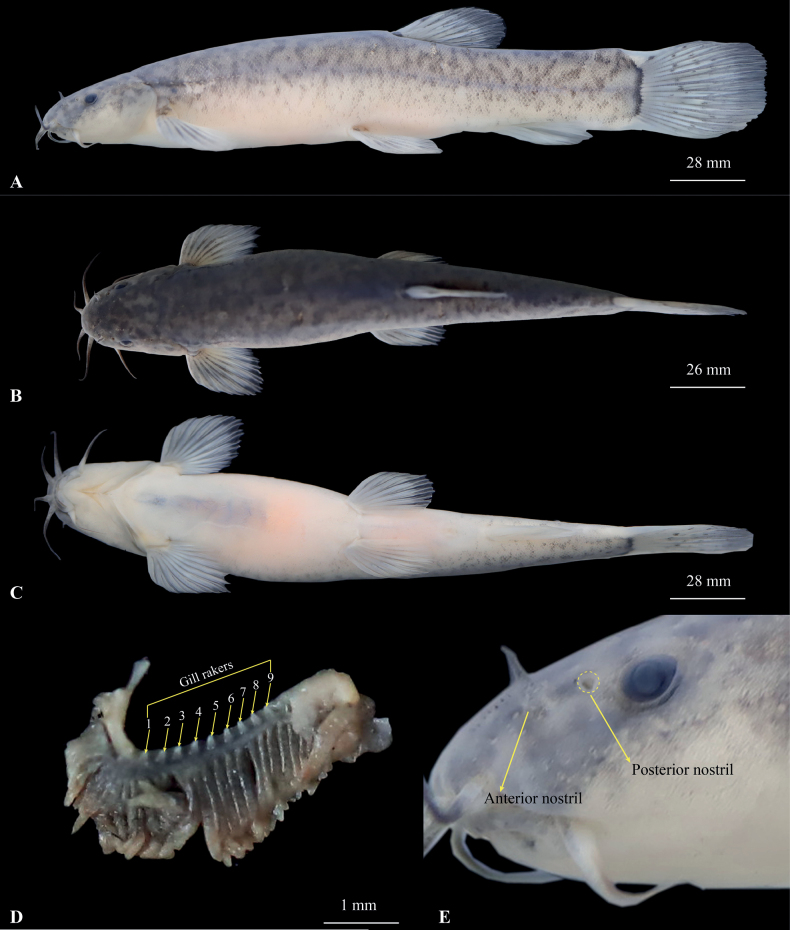
Morphological characteristics of the holotype GZNU20230216001 of *Oreonectesdamingshanensis* sp. nov. in preservative (10% formalin) **A** lateral view **B** dorsal view **C** ventral view **D** gill raker **E** enlarged anterior and posterior nostrils.

**Figure 5.**
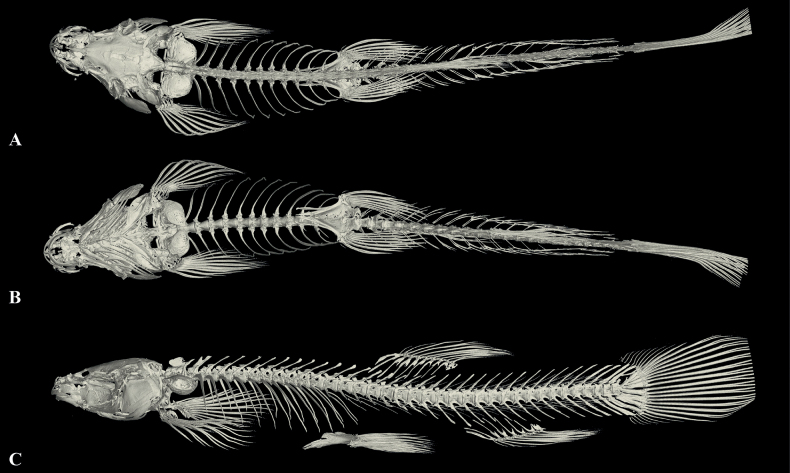
The three-dimensional reconstructed model of the skeleton of *Oreonectesdamingshanensis* sp. nov. (paratype GZNU20230216021, standard length 57.0 mm) **A** dorsal view **B** ventral view, and **C** latera view. Caudal fin slightly broken.

**Figure 6.**
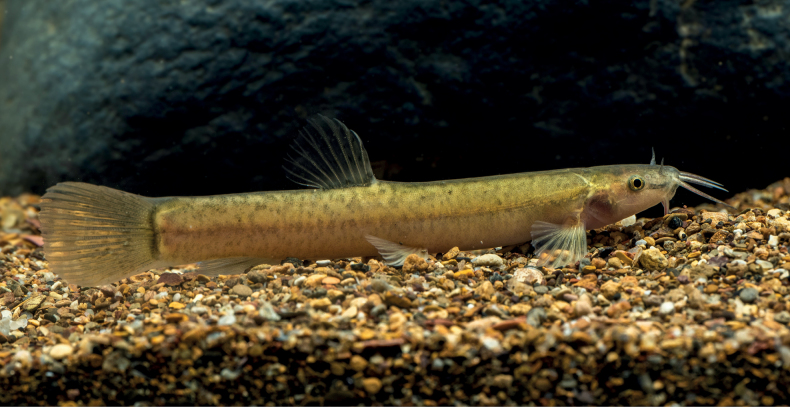
Live paratype of *Oreonectesdamingshanensis* sp. nov.

Figs 4 , 5 , 6 ; Appendix 3 : Table A1

#### Chresonymy.

*Oreonectesplatycephalus* (Günther, 1868): Wang 2022 (Guangxi, China); Luo et al. 2023 (Damingshan Mountains, Shanglin County, Guangxi, China).

#### Material.

***Holotype*.** GZNU20230216001, 98.9 mm total length (TL), 81.8 mm standard length (SL), collected by Jing Yu on February 16, 2023, in Waminggu Scenic Area, Leping Village, Guling Town, Mashan County, Guangxi Zhuang Autonomous Region, China (23.60818443°N, 108.29426408°E; ca. 234 m a.s.l.). ***Paratypes*.** Twenty-four specimens from the same locality as the holotype: GZNU20230216002–216025, 46.2−70.7 mm SL, collected by Jing Yu and Tao Luo on February 16, 2023. Five specimens from Damingshan Mountains, Shanglin County, Guangxi: GZNU2020011505–011509, 59.2−76.7 mm SL, collected by Yali Wang and Tao Luo on February 16, 2021.

#### Etymology.

The species epithet *damingshanensis* refers to the type locality, located within the Damingshan Mountains, Guangxi, China. The suggested English name is the Damingshan Mountains loach, and the Chinese name is Dà Míng Shān Lıˇng Qiū (大明山岭鳅).

#### Diagnosis.

*Oreonectesdamingshanensis* sp. nov. is assigned to the genus *Oreonectes* based on molecular phylogenetic analyses and the following characteristics, which are diagnostic for this genus: (1) anterior and posterior nostrils narrowly separated; (2) lips smooth, with furrows; (3) barbel-like elongation of anterior nostrils longer than depth of nostril tube; and (4) caudal fin rounded, dorsal fin with 6 or 7 branched rays (Du et al. 2023).

*Oreonectesdamingshanensis* sp. nov. can be distinguished from all other congeners by the following combination of characters: (1) body pigmentation present; (2) eyes normal, diameter 6.5–17.1% of head length; (3) caudal fin rounded, with irregular black markings; (4) body completely covered with fine scales except for the head; (5) lateral line incomplete, with 14 or 15 pores, last lateral line pore reaching above the tip of pectoral fin; (6) dorsal-fin rays, iii-7; (7) pectoral-fin rays, i-9; (8) anal-fin rays, iii-5; (9) pelvic-fin rays, i-7; (10) 14 branched caudal-fin rays; (11) tip of ventral fin extended backward, not reaching the anus; (12) dorsal fin origin posterior to the pelvic-fin origin; (13) inrostral barbel extending backward and not reaching the anterior margin of the eye; (14) tip of maxillary barbel not reaching the posterior margin of the gill cover; and (15) vertebrae 4 + 34.

#### Description.

Body elongated and cylindrical, with the insignificant depth decreasing from dorsal-fin origin to caudal-fin base. Head short, length 13.3 mm, slightly depressed and flattened, width greater than depth (head width/head depth = 1.3). Snout round, oblique and flat, length 38.0–45.3% of head length (HL). Mouth inferior, curved, upper and lower lips smooth, lower lip with a V-shaped median notch. Three pairs of barbels are long: inrostral barbel length 25.9–38.3% of HL, extending backward and not reaching the anterior margin of eye; outrostral barbel length 42.4–62.4% of HL, extending backward beyond the posterior margin of the eye. Maxillary barbel length 36.0–56.6% of HL, tip of maxillary barbel not reaching to posterior margin of gill cover. Anterior and posterior nostrils narrowly separated, length 33.5–84.2% of eye diameter. Anterior nostril tube short, with an elongated short barbel-like tip. Eyes present, normal, diameter of 6.5–17.1% HL. Gill opening small; gill rakers not developed, nine inner gill rakers on the first gill arch (*n* =1) (Fig. 4D).

Dorsal-fin rays iii-7, pectoral-fin rays i-9, pelvic-fin rays i-7, anal-fin rays iii-5, 14 branched caudal fin rays. Dorsal fin short, length 15.8–22.6% of SL, distally margin round, origin posterior to pelvic-fin insertion, situated slightly posterior to two-thirds the distance between snout tip and caudal-fin base. Pectoral fin short, length 15.2–19.5% of SL, tip of pectoral fin extending backward to ~56.7% of the distance between the origin of pectoral and pre-pelvic fins. Pelvic fin length 13.1–17.2% of SL, tips of pelvic fin not reaching anus, distance between tips of pelvic fin and anus 2.0 times the eye diameter. Anal fin long, length 15.3–179.7% of SL, tips of anal fin not reaching caudal-fin base, distance between tips of anal fin and anus 0.64 times the eye diameter. Caudal fin rounded, caudal peduncle length 9.3 mm, without adipose crests along either dorsal or ventral sides. Vertebrae 38 comprising of 4 Weberian + 22 abdominal + 11 caudal + 1 compound centrum (Fig. 5).

Except for the head, body completely covered by scales. Lateral line incomplete, exceeding tip of pectoral fin but not reaching base of caudal fin, with 14 or 15 pores, last lateral line pore reaching above the tip of pectoral fin. Cephalic lateral-line system, with eight supraorbital, 5 + 8 infraorbital canal pores, three supratemporal canal pores, with eight preoperculo-mandibular canal pores. Two chambers of air-bladder, posterior chamber developed, filling the body cavity and connected with anterior chamber by a long, slender tube. Lateral wall of the bony capsule of the swim bladder is membranous and closed posteriorly (Fig. 5).

#### Coloration.

In life, body pale yellow-brown overall, dark brown between anterior margin of eyes to outrostral barbel, brown lateral stripe on flank of the body, irregular black spots present on dorsal and lateral surfaces and caudal fin, black at base of caudal fin, pale brown on each fin (Fig. 6). In 10% formalin, the body color was dark brown (Fig. 4).

#### Comparisons.

Comparative data of *Oreonectesdamingshanensis* sp. nov. with the five known species within the genus *Oreonectes* are given in Table 1.

*Oreonectesdamingshanensis* sp. nov. can be distinguished from *O.luochengensis* by nine branched pectoral-fin rays (vs 11 or 12), lateral line pores 14 or 15 (vs 6–13), caudal fin rounded, with irregular black markings (vs truncated, without irregular black markings), body covered with scales except for the head (vs degraded, body scales hidden under the skin), and vertebrae 4 + 34 (vs 4 + 32 /35).

*Oreonectesdamingshanensis* sp. nov. can be distinguished from *O.guilinensis* by dorsal-fin rays (iii, 7 vs ii, 6), seven branched pelvic-fin rays (vs 6), lateral line pores 14 or 15 (vs 4–6), black at base of caudal fin (vs absence), maxillary barbel extending backward, not reaching to the posterior margin of the gill cover (vs reaching to posterior margin of the eye), and tip of pelvic fin not reaching to anus (vs reaching to anus).

*Oreonectesdamingshanensis* sp. nov. can be distinguished from *O.guananensis* by nine branched pectoral-fin rays (vs 10 or 11), dorsal fin origin posterior to pelvic-fin origin (vs opposite to pelvic-fin origin), maxillary barbel extending backward and not reaching to posterior margin of gill cover (vs reaching to the gill cover), lateral line pores 14 or 15, last lateral line pore reaching above the tip of pectoral fin (vs 7–13, last lateral line pore not reaching above the tip of pectoral fin), caudal fin with irregular black markings (vs without irregular black markings), vertebrae 4 + 34 (vs 4 + 32), relatively small eye diameter (1.6 ± 0.3 mm vs 5.8 ± 1.9 mm, *p*-value = 0.00, Table 4) and some significant morphological differences (*p*-values < 0.05), including head depth (4.7 ± 0.8 mm vs 3.1 ± 1.0 mm), head width (9.7 ± 1.3 mm vs 7.0 ± 1.8 mm), mouth width (7.2 ± 1.0 mm vs 4.7 ± 1.5 mm), interorbital distance (5.6 ± 0.9 mm vs 1.7 ± 0.4 mm), pectoral-fin base length (2.4 ± 0.4 mm vs 1.8 ± 0.5 mm), pelvic-fin base length (2.3 ± 0.4 mm vs 1.2 ± 0.5 mm), and anal-fin base length (5.2 ± 0.6 mm vs 4.2 ± 0.8 mm).

*Oreonectesdamingshanensis* sp. nov. differs from *O.platycephalus* by having seven branched dorsal-fin rays (vs 8 or 9), nine branched pectoral-fin rays (vs 11), seven branched pelvic-fin rays (vs 8), anal-fin rays (iii, 5 vs ii, 6 or 7), nine inner gill rakers on the first gill arch (vs 14 or 15), and maxillary barbel extending backward, not reaching to the posterior margin of the gill cover (vs reaching to the posterior margin of the eye).

*Oreonectesdamingshanensis* sp. nov. can be distinguished from *O.polystigmus* by having seven branched pelvic-fin rays (vs 6), nine branched pectoral-fin rays (vs 10), seven branched pelvic-fin rays (vs 6), anal-fin rays (iii, 5 vs ii, 5), lateral line pores 14 or 15 (vs 6–8), nine inner gill rakers on first gill arch (vs 13 or 14), tip of pelvic fin not reaching to anus (vs reaching to anus), maxillary barbel extending backward, not reaching the posterior margin of the gill cover (vs reaching the pectoral-fin origin), and vertebrae 4 + 34 (vs 4 + 32) . In addition to differences in each fin, the new species also differed significantly from *O.polystigmus* in a number of measurable characters. These included larger head length (13.3 ± 1.7 mm vs 11.6 ± 1.0 mm), head width (9.7 ± 1.3 mm vs 7.7 ± 0.3 mm), mouth width (4.7 ± 0.8 mm vs 3.1 ± 0.4 mm), eye diameter (1.6 ± 0.3 mm vs 0.9 ± 0.2 mm), dorsal-fin length (11.4 ± 1.4 mm vs 9.5 ± 0.3 mm), pelvic-fin length (9.6 ± 1.3 mm vs 7.5 ± 0.4 mm), and anal-fin length (10.4 ± 1.2 mm vs 8.1 ± 0.4 mm) (*p*-value < 0.05; Table 4).

#### Distribution, habitat, and populations.

Based on current field surveys, the new species *Oreonectesdamingshanensis* sp. nov. has only been found in streams in the Damingshan Mountains of Mashan and Shanglin counties in the Guangxi Zhuang Autonomous Region of China belonging to the Hongshuihe River basin of the Pearl River system. The new species is easy to collect in the streams of the Damingshan Mountains area where it has a large population.

### ﻿Key to species of the gneus *Oreonectes*

**Table:** 

1	Body pigmentation absent, caudal fin truncated	***O.luochengensis***
–	Body pigmentation present, caudal fin rounded	**2**
2	Caudal fin with irregular black markings	**3**
–	Caudal fin without irregular black markings	**4**
3	Seven branched dorsal-fin rays, tip of pelvic fin not reaching anus	***O.damingshanensis* sp. nov.**
–	Six branched dorsal-fin rays, tip of pelvic fin reaching anus	***O.guilinensis***
4	Dorsal fin origin opposite to pelvic-fin origin	***O.guananensis***
–	Dorsal fin origin posterior to pelvic-fin origin	**5**
5	Tip of pelvic fin not reaching anus, maxillary barbels reaching posteriorly to the posterior margin of eye	***O.platycephalus***
–	Tip of pelvic fin not reaching anus, maxillary barbels reaching posteriorly to the pectoral-fin origin	***O.polystigmus***

## ﻿Discussion

Both morphological (see comparisons above) and mitochondrial genetic differences, i.e., forming a distinct lineage with an uncorrected *p*-distance of 6.1% from *O.polystigmus*, support the validity of *Oreonectesdamingshanensis* sp. nov., a new species found in the mountain streams of the Damingshan Mountains, Nanning City, Guangxi, China (Fig. 1). With the addition of this new species, the true genus *Oreonectes* now comprises six species, namely *Oreonectesdamingshanensis* sp. nov., *O.guananensis*, *O.guilinensis*, *O.luochengensis*, *O.platycephalus*, and *O.polystigmus*. In the phylogenetic tree, the new species *Oreonectesdamingshanensis* sp. nov. from Damingshan Mountains is not fully resolved and supported, although it forms a sister clade with (*O.guilinensis* + *O.platycephalus*) (Fig. 2). This may be related to the number and length of molecular markers and the number of species. In the phylogenetic tree reconstructed by Luo et al. (2023) based on the mitochondrial genome and seven nuclear genes, *O.platycephalus* (No. 27, i.e., *Oreonectesdamingshanensis* sp. nov.) from the Damingshan Mountains was considered to be a sister species of *O.polystigmus*, which was strongly supported by the BI tree but not fully resolved in the ML tree. This may be related to the absence of *O.guilinensis*. Therefore, the inclusion of a sufficient number of sampled species and molecular markers in future studies may help to resolve interspecific phylogenetic relationships within the genus *Oreonectes*.

The widely distributed species *O.platycephalus* Günther, 1868 may be a complex containing multiple cryptic species. *O.platycephalus* was originally described from specimens collected from small streams near the top of Hong Kong Mountains, the type locality (Günther 1868), and it has been extensively recorded from various tributary areas of the Pearl River Basin (the Luofu Mountains, the Baiyun Mountains and eastern Guangdong, and Jinxiu, Rong’an, and Zhaoping counties in Guangxi), including areas in northern Vietnam (Fig. 1) (Zhu 1989; Kottelat 2001; Zhang et al. 2016). The wide geographical distribution, morphological similarity, and classification based on morphological characteristics of species from the same basin may lead to different geographical populations of the species being identified as a single species, resulting in an underestimation of species diversity. The combination of mitochondrial differences and morphological characteristics of *O.guilinensis* supports this hypothesis (Huang et al. 2020). Furthermore, the small-bodied, widely distributed species of *Oreonectes* may rapidly form new species due to the geographical isolation of water systems, as shown in Fig. 1 and the phylogenetic tree (Fig. 2). Therefore, we recommend that a comprehensive and systematic survey (especially concerning the grey shaded area in Fig. 1) of *O.platycephalus* be carried out to assess its species diversity.

In the newly described species *Oreonectesdamingshanensis* sp. nov. and the other five species, *O.guananensis* and *O.luochengensis* are the most northerly distributed and have adjacent distributions and phylogenies, followed by *O.polystigmus* and finally *O.guilinensis* and *O.platycephalus*. By combining the positions of these species in the phylogenetic tree, we propose the hypotheses of “neighboring and closely related” and “northern Guangxi origin with southward dispersal”, i.e., within the genus *Oreonectes*, the shorter the distance between species, the more closely related they are in the phylogenetic tree, and the earlier the species formed at the northern tip of their distribution. These “neighboring and closely related”, “microlocalism”, and “north to south” patterns of geographic distribution and phylogeny suggest that *Oreonectes* is a good candidate for speciation and biogeographic studies in the Pearl River Basin. To test this hypothesis, future studies could focus on population genetics and biogeographic study of the *O.platycephalus* complex.

## Supplementary Material

XML Treatment for Oreonectes damingshanensis

## ﻿Additional information

### Author ORCIDs

Jing Yu https://orcid.org/0009-0004-3629-3826

Tao Luo https://orcid.org/0000-0003-4186-1192

Chang-Ting Lan https://orcid.org/0009-0007-2381-3601

Jia-Jun Zhou https://orcid.org/0000-0003-1038-1540

Huai-Qing Deng https://orcid.org/0000-0002-6645-2291

Ning Xiao https://orcid.org/0000-0002-7240-6726

Jiang Zhou https://orcid.org/0000-0003-1560-8759

### Data availability

All of the data that support the findings of this study are available in the main text or Appendices.

## ﻿Appendix 1

Specimens examined in this work.

*Oreonectesguananensis* (*n* = 5): China: Guangxi: Huanjiang County: Changmei Town (type locality): GZNU2020050201–050205.

*Oreonectesluochengensis* (*n* = 5): China: Guangxi: Luocheng County: Tianhe Town (type locality): GZNU2017001–005.

*Oreonectesplatycephalus* (*n* = 6): China: Guangdong: Shenzhen City: Wutong Mountain: GZNU2020011510–011515.

*Oreonectespolystigmus* (*n* = 4): China: Guangxi: Guilin City: Yanshan District: Dabu Town (type locality): GZNU201908004–908007.

## ﻿Appendix 3

**Table A1.** Measurements of the five specimens of *Oreonectesdamingshanensis* sp. nov. and other species. All units in mm. *designates the holotype.

ID	Species	Voucher number	Total length	Standard length	Body depth	Body width	Head length	Head depth	Head width	Distance between anterior nostrils	Distance between posterior nostrils	Distance between anterior and posterior nostrils	Snout length	Upper jaw length	Lower jaw length	Mouth width	Eye diameter	Interorbital distance	Predorsal length	Dorsal-fin base length	Dorsal-fin length	Pectoral-fin length	Pectoral-fin base length	Prepectoral length	Pelvic-fin length	Pelvic-fin base length	Prepelvic length	Anal-fin length	Anal-fin base length	Preanal length	Caudal peduncle length	Caudal peduncle depth	Maxillary barbel length	Inrostral barbel length	Outrostral barbel length
1	*O.damingshanensis* sp. nov.	GZNU20230216001	98.9	81.8	14.8	10.2	16.6	9.5	13.4	6.5	6.5	1.1	7.1	4	3.3	5.9	1.9	7.4	48.7	8.6	14.7	13	3.5	15.7	12.3	3.3	41.4	13.6	6.5	61.3	14.6	9.5	8.1	5.2	9.7
2	*O.damingshanensis* sp. nov.	GZNU20230216002	77.8	63.5	10.9	7.2	13.6	7.9	9.5	3.6	4.9	1.1	5.4	3.4	2.2	4.7	1.6	5.8	38.6	7.2	13.8	11.5	2.4	13.5	10.9	2.5	31.5	11.5	5.7	47.5	10.3	6.7	4.9	3.9	7.4
3	*O.damingshanensis* sp. nov.	GZNU20230216003	75.3	61.8	9	6.5	12.5	6.9	9.7	3.5	4.7	1	5.1	3.1	2.6	5	1.9	5.3	37.4	6.7	11.9	11.3	2.8	12.7	10.2	2.6	30	10.7	5.5	46.1	9	7	5.7	4.4	6.2
4	*O.damingshanensis* sp. nov.	GZNU20230216004	86.9	70.7	10.5	8.1	16.2	8.8	12.6	5.1	6.1	1.5	7	4.1	3.2	6.4	1.9	6.7	42.4	7.7	13.5	13.2	2.8	16.1	11.8	3	36	11.4	5.6	53.3	11.8	7.5	7	4.2	8.8
5	*O.damingshanensis* sp. nov.	GZNU20230216005	63.7	52.5	7.7	5.8	11.8	6.8	8.6	3.4	4.4	1.3	4.7	2.9	2.1	4.1	1.5	4.9	32.4	5.1	10.3	8.2	2.2	11.6	7.7	2.1	27.4	8.5	4.9	40.3	7.9	5.3	4.9	3.9	6.6
6	*O.damingshanensis* sp. nov.	GZNU20230216006	73	59.9	8.7	6.2	13.3	7.3	9.8	4.3	5	0.9	5.2	3.2	2.8	4.8	1.5	5.6	36.2	6.6	11.7	10.3	2.4	12.9	10	2.2	30.7	10.7	5.2	45.3	9.7	6.4	6.5	5	8.3
7	*O.damingshanensis* sp. nov.	GZNU20230216007	81.3	67.6	10.8	7.8	14.8	7.6	10.8	4.5	5.2	1	6.1	3.9	3.1	5.3	1.9	6.2	42.1	6.2	12.7	10.5	2.6	14.5	10.3	2.5	34.3	11.1	4.9	50.7	9.7	7.2	5.9	5.2	8.2
8	*O.damingshanensis* sp. nov.	GZNU20230216008	75	62.2	8.8	7.2	13.2	7.5	9.5	3.1	4.5	0.9	5	3.1	2.4	5	1.5	4.6	37.6	6.5	10.7	9.8	2.5	13.6	9.1	2	30.7	10.5	5.2	46.9	10.1	6.7	6.7	4.6	7.5
9	*O.damingshanensis* sp. nov.	GZNU20230216009	79.2	66.4	9.8	7.3	14.2	7.2	9.6	4	5	0.9	5.4	3.2	2.6	5.3	2	5.8	39.8	6	11.8	11.1	2.4	13.8	10.2	2.5	34.1	10.9	5.8	50.2	10.2	6.7	5.7	4.7	6.7
10	*O.damingshanensis* sp. nov.	GZNU20230216010	77.2	62.8	10.3	7.4	14.4	8.1	11	4.3	5.1	1.1	6.1	3.8	2.7	5.7	1.7	6	38.6	6.4	11.2	10.7	2.7	14.3	9.8	2.2	32.3	10.6	5.4	48.9	9.3	7.1	5.5	5.2	7.1
11	*O.damingshanensis* sp. nov.	GZNU20230216011	73	59.6	8.9	7	13.1	6.9	9.8	4	4.6	0.8	5.6	3.2	2.4	4.9	1.7	5.3	37.4	5.9	11.1	10	2.4	12.7	10	2.2	30.7	10.2	5.1	45	8	6.7	5.4	4.7	6.9
12	*O.damingshanensis* sp. nov.	GZNU20230216012	74.3	61.3	10.2	7	13.2	7.4	9.8	4.1	4.5	0.8	5.5	3.1	2.6	4.6	1.7	5.7	38.3	5.6	11	10.6	2.7	13	9.4	2.4	31	9.4	4.7	46.8	9.8	6.7	6	4.1	6.4
13	*O.damingshanensis* sp. nov.	GZNU20230216013	73.3	60.8	10.2	6.9	14.2	8.1	11.1	4.2	5.1	0.9	6.1	3.5	2.9	5.4	1.8	6	37.6	6.5	12	10.2	2.6	13.9	9.8	2.5	31.8	10.6	5.7	46.1	9.3	6.7	6.6	4.1	7.4
14	*O.damingshanensis* sp. nov.	GZNU20230216014	64.6	52.9	8.2	5.9	12	6	8.2	3.4	4.1	0.6	4.7	2.9	2.4	4.4	1.6	4.7	31.8	5.3	10.1	10.3	2.3	11.9	8.5	2.1	28.4	9.8	4.5	40.1	8.6	5.7	5.8	4.3	6.3
15	*O.damingshanensis* sp. nov.	GZNU20230216015	63.4	52.8	8	5.7	12.1	6.6	8.1	3.6	4.1	0.8	4.7	3.1	2.6	4.7	1.6	4.8	31	5	8.8	8.3	1.9	11.2	6.9	2.3	26.5	8.9	4.6	39.9	8	5.9	4.6	3.9	5.5
16	*O.damingshanensis* sp. nov.	GZNU20230216016	77.8	64.1	9.7	8	14.1	7.9	10.7	4.5	4.9	0.6	5.8	3.1	2.6	5.2	1.9	6	38.7	6.3	12.5	10.6	2.5	13.7	10	2.6	31.8	10.7	5.5	49.6	9.8	6.9	7	5.4	8
17	*O.damingshanensis* sp. nov.	GZNU20230216017	72.1	60.5	10.3	7.2	13.6	7.3	9.7	4.6	5	1	5.4	3.2	2.6	5.2	1.7	6	36.4	6.1	10.9	9.2	2.3	12.8	9.4	2.6	30.4	9.7	5.1	46.2	9.8	7.2	6.1	4.7	7.3
18	*O.damingshanensis* sp. nov.	GZNU20230216018	72.6	59.5	9.1	6.4	14	7.5	10.7	4.1	4.6	0.7	5.7	3.4	2.4	5	2	5.5	35.9	5.8	10.6	10	2.4	13.7	9.3	2.6	31	10	5.6	45.6	8.1	6.5	5.5	4.2	6.9
19	*O.damingshanensis* sp. nov.	GZNU20230216019	66.6	54.3	7.8	5.8	12.8	6.9	9.7	4.1	4.2	0.7	5.7	3.1	2.6	5	1.6	5.3	32.5	5.9	10.4	9.4	2.4	12.7	8.7	2.1	27.7	10.3	4.9	40.5	8.4	6.3	5.1	3.7	6.6
20	*O.damingshanensis* sp. nov.	GZNU20230216020	78.9	64.1	8.9	6.3	13.7	7.5	10.6	3.5	4.7	1	5.9	3.6	2.8	5.7	1.9	5.4	37.5	6.6	11.9	10.2	2.6	14.4	10	2.4	33.3	10.8	5.6	49.2	10.3	7.3	6.6	4.5	6.6
21	*O.damingshanensis* sp. nov.	GZNU20230216021	69.6	57	9.4	6.6	12.5	7.2	9.7	3.8	4.3	0.8	5.1	3.2	2.5	4.9	1.8	5.4	33.5	6.1	11.4	9.3	2.6	12.2	8.5	2.4	29.1	9.7	4.8	42	9.3	6.8	6.2	4.6	6.7
22	*O.damingshanensis* sp. nov.	GZNU20230216022	59.1	47.8	7.1	5	10.6	5.5	7.7	3	3.6	0.7	4.5	3.1	0.3	3.7	1.4	4.5	27	5.5	10.8	8.3	2	10.1	8.1	1.5	23.8	9.4	4.2	35.2	8.4	4.9	6	3.9	5.4
23	*O.damingshanensis* sp. nov.	GZNU20230216023	59.7	48.4	7.2	5.4	10.5	5.9	8.3	3.2	3.8	0.7	4.2	2.9	2.4	4.1	1.8	4.4	29.4	5.1	10	8.3	1.9	10.9	7.9	2	25.2	9.1	4.4	36.8	6.8	5	4.9	4	5.9
24	*O.damingshanensis* sp. nov.	GZNU20230216024	60.5	48.9	7	5.3	10.8	5.7	8	3.4	4	0.7	4.5	3.1	2.3	4.1	1.7	4.5	27.8	5.6	10.4	8.7	1.9	10.5	7.7	1.9	24.1	9.4	5.2	35.8	6.9	5.2	4.4	4.1	5.6
25	*O.damingshanensis* sp. nov.	GZNU20230216025	55.7	46.2	7	5	10.2	5.4	7.8	2.8	3.4	0.6	4.4	2.5	2.1	3.9	1.6	4.4	27.9	4.9	8.8	8.1	1.8	9.8	7.7	1.5	23.8	8.3	4.4	34.5	6.4	4.9	4.6	3.9	5.8
26	*O.damingshanensis* sp. nov.	GZNU2020011505	91	76.7	12.5	8.3	16.5	8.8	9	3.8	6.1	1.1	6.9	3.6	2.7	4.8	1.3	7.7	45.9	9.1	13.9	12.2	3	17.3	12.2	2.5	40.6	13.3	7.2	57.5	10.5	8	6.6	5.6	7.1
27	*O.damingshanensis* sp. nov.	GZNU2020011506	79	64.2	9.6	8	13.9	7	9.3	3.2	4.8	0.9	5.5	2.2	2.1	3.3	0.9	5.6	37.1	6.8	13	10	2.3	14.4	10	2.1	33.1	10.8	4.9	49.1	11	7.9	5	4.7	7.4
28	*O.damingshanensis* sp. nov.	GZNU2020011507	80.3	67.8	11.5	4.6	15	7.4	9	3.7	5.1	0.8	6.8	2.7	2	4.4	1	6.9	39.3	6.7	10.7	11	2.6	14.5	11	2.6	34.3	10.8	5.5	51.6	9.1	7.1	6.6	5.2	6.9
29	*O.damingshanensis* sp. nov.	GZNU2020011508	71.9	59.2	11	7.1	13.9	7.4	9.9	3.2	4.6	0.9	5.9	2.6	1.8	2.5	1	6.1	39.8	5.8	11.8	9.8	2.2	14.9	9.8	2.4	35.5	10.8	5.4	52.7	9	6.6	5.2	4.6	5.9
30	*O.damingshanensis* sp. nov.	GZNU2020011509	72.8	61.4	7.9	7	12.1	6.1	8.7	3.1	5.2	0.8	5.1	2.8	2.1	3.6	1.7	6.9	36.4	5.1	10.8	9.9	2.6	12.5	9.9	2.4	30.7	10	4.7	46.1	9.2	7	6	3.5	5.3
31	*O.platycephalus*	GZNU2020011510	52.2	51.1	6.3	4.8	10.5	5.8	7.6	2.7	4.5	0.9	3.9	1.7	1.5	2.9	1.7	5.7	31.3	4.1	9	8.6	2	10.9	8.8	1.9	26.1	8.6	4.1	38.2	8.6	6	4.8	3.2	5.1
32	*O.platycephalus*	GZNU2020011511	52.3	41.8	6.4	4.7	9	4.3	6.2	1.9	3.4	0.9	3.3	1.8	1.4	2.4	0.8	4.6	24.9	3.9	7.3	7.8	1.9	9	8.2	1.9	21.5	7.6	3.4	32.1	6.5	4.9	4.3	3.3	4.4
33	*O.platycephalus*	GZNU2020011512	53.5	44.4	6.3	3.6	9	4.6	6.5	2	3.9	0.8	3.8	1.8	1.4	2.9	0.8	4.6	27	3.5	7.3	6.9	1.8	9.5	7.3	1.4	21.1	7.6	3.5	33.5	7.3	4.6	1.8	3	4.3
34	*O.platycephalus*	GZNU2020011513	53.5	44.4	6.3	3.6	9	4.6	6.5	2	3.9	0.8	3.8	1.8	1.4	2.9	1	4.6	27	3.4	7.3	6.9	1.8	9.5	7.3	1.4	21.1	7.6	3.5	33.5	7.3	4.6	3.6	3	4.3
35	*O.platycephalus*	GZNU2020011514	41	33.1	4.6	2.8	6.6	2.9	4.5	1.7	2.3	0.6	1.9	1.2	0.9	1.6	0.5	3.7	19.7	2.1	4.9	5.1	1.3	7	5.3	1.2	16.8	4.8	2	24.4	5.6	3.5	2.7	1.5	3.4
36	*O.platycephalus*	GZNU2020011515	85.5	73.6	12.2	7.6	16.7	6.5	9.1	3.8	6.1	1.5	7	3.5	2.4	4.1	1.9	8.2	43.1	6.4	9.9	10.8	2.7	17.6	11.1	2.6	39.3	8.7	5.2	56	11.1	7.6	6.2	5.5	8.1
37	*O.guananensis*	GZNU2020050201	85.8	70	9	6.7	16	6.2	8.6	3.7	6.6	1.4	6.2	3.7	2.5	4.4	7.7	2	40.9	6.2	13.2	11.5	2.1	16.7	9.7	1.4	39.3	12	5	55.4	8.9	8.1	8.9	4.2	6.4
38	*O.guananensis*	GZNU2020050202	64.3	53.9	7.9	4.6	11.5	3.3	6	2.6	4.3	1.4	4.4	2.5	1.9	2.6	5.3	2.1	31.4	4.7	9.7	5.3	1.8	12.1	6.1	0.8	28.6	7.8	3.5	40.6	7.6	5.5	7.2	4.3	8.8
39	*O.guananensis*	GZNU2020050203	89	73.8	9.8	6.9	16.8	6.5	8.4	4	5.4	1.3	6.8	3.2	2.8	3.8	7.8	1.1	42.5	7.3	14.9	11	2.3	17.2	10.2	1.9	40.4	11.5	5.1	57.2	9	7.6	7.5	7	9.1
40	*O.guananensis*	GZNU2020050204	50.5	40.6	5.7	4.2	8.9	3.5	4.3	1.8	3.7	1.2	3.6	1.7	1.5	2	4.3	1.6	24.8	2.5	7.2	6.6	1.1	9.6	5.5	0.8	22.9	6.7	3.4	32.7	7.1	4	4.9	3.4	5.5
41	*O.guananensis*	GZNU2020050205	52.6	45	4.5	5.8	5.1	4.2	7.9	2.2	2.6	1.8	4.6	2	1.9	2.8	3.7	1.6	22.5	4.5	9.5	9.3	1.8	12.1	8.5	0.9	23.3	7.1	3.8	35.7	8.1	3.4	3.6	2.2	4.2
42	*O.anophthalmus*	GZNU20190810	32	27.2	3.2	2.9	6	2.5	4	0	0	0	-	1.2	1.1	1.9	0	0	17.9	2.5	4.4	4	20.3	4.4	3	0.8	15	3.6	1.9	20.3	4.6	2.4	3.4	2	3.5
43	*O.anophthalmus*	GZNU20190811	26.8	22.1	2.7	1.9	5.2	2	3.1	0	0	0	-	1.5	1.1	0.3	0	0	12.6	2.3	3.1	3.2	0.9	5.5	2.9	0.8	13.2	3.7	1.5	16.8	3.5	1.6	1.7	0.8	2.1
44	*O.anophthalmus*	GZNU20190812	26.4	23.1	2.8	2	5.3	2.3	3.4	0	0	0	-	1.1	1.1	1.8	0	0	14.6	1.8	2.9	3	0.7	5.7	2.3	65	12.7	2.7	1.5	17.3	3	2.1	1.1	0.7	1.7
45	*O.polystigmus*	GZNU201908004	66.4	55.5	9.5	8.7	11.9	7.8	8.1	2.8	5.1	1.1	4.4	2.3	1.9	2.9	0.8	6.3	33	4.4	9.7	9.3	2.4	12.6	8	1.7	30.3	7.6	3.9	44.7	9.9	5.9	6.9	4.4	6
46	*O.polystigmus*	GZNU201908005	61.5	51.2	6	5.5	10.2	5.7	7.4	2.4	4.2	1.2	10.7	2.1	1.5	2.6	0.8	5	29.6	5	9.5	8.8	2.3	11.1	7.1	1.7	26.1	8	3.9	38.1	9	4.4	5.2	5	8.1
47	*O.polystigmus*	GZNU201908006	66.9	54.6	8.3	6.1	11.6	6.3	7.6	2.6	4.3	1.2	4.6	2.2	1.8	3.3	1	6.2	31.8	4.7	9	9	1.6	11.5	7.2	1.4	29.8	8.1	3.9	43.4	9.6	5.1	5.6	4.4	6.7
48	*O.polystigmus*	GZNU201908007	66.4	55.3	11.4	7.5	12.5	7.3	7.6	2.9	4.3	1	4.7	2.6	1.6	3.6	1.1	6.8	33.2	5.9	9.6	9.3	2.7	12.8	7.8	1.6	29.1	8.6	5	43	6.5	5.8	6	6.1	7
49	*O.luochengensis*	GZNU2017001	80.6	65.6	9.3	9.4	13.2	6.5	7.6	3.3	3.1	1.4	5.5	2.5	2.3	4	6.6	2.1	38.2	6.2	11	11.9	2.2	15.1	8.9	1.7	36.2	9.4	4.3	53.6	7.4	6.1	5.2	4.5	7.4
50	*O.luochengensis*	GZNU2017002	80.3	66.2	8.7	7	13	7.3	7.4	3	3.8	1.2	4.5	2.9	2.4	3.8	6.4	2.2	37.5	6.2	12.3	11.4	2.8	13.3	8.9	2	35.1	9.9	5.7	30.7	7.3	6.4	6.7	4.7	6.5
51	*O.luochengensis*	GZNU2017003	61.7	51.7	7.3	6.3	10.6	5.6	6.1	2.3	3.3	1.1	7.7	2.5	1.8	3.1	5.7	1.6	29.8	4.1	9.5	9.2	1.6	11.4	11.4	1.6	28.5	8.4	3	40.7	8.1	5.7	5.1	3.7	5.1
52	*O.luochengensis*	GZNU2017004	69.6	57.7	6.2	6.3	11.9	6.8	7	2.7	4.8	1.2	4.6	2.9	2.5	3.6	5.9	1.7	31.8	5.1	10.5	10	1.8	12.4	7.8	1.7	31.3	7.8	2	43.7	7.2	4.7	6.8	3.6	6.2
53	*O.luochengensis*	GZNU2017005	68.3	56.4	9	6.9	11.9	6.4	5.6	3.2	4.4	1.3	4.1	2.3	2.1	3.8	5.5	1.8	33.9	4.4	9.4	7.9	1.7	12.4	6.6	1.6	29.9	7.5	4.3	42.5	6.8	5.1	5.2	3.6	4.5

## ﻿Appendix 4

**Table A2.** Results and percentage of variance explained by principal component and discriminant function analyses.

Morphometric characters	PC 1	PC 2	CAN1	CAN2
Total length	0.98	0.09	2.16	1.52
Standard length	0.98	0.13	-1.32	2.51
Body depth	0.91	0.16	0.66	3.09
Body width	0.89	0.10	-1.12	0.33
Head length	0.98	0.04	22.67	-4.69
Head depth	0.95	-0.03	-1.73	2.03
Head width	0.93	-0.23	0.06	-1.33
Distance between anterior nostrils	0.89	-0.33	0.83	0.33
Distance between posterior nostrils	0.93	0.23	2.20	0.78
Distance between anterior and posterior nostrils	0.48	0.62	0.11	1.50
Snout length	0.81	0.13	-1.95	0.66
Upper jaw length	0.87	-0.36	-3.74	-1.86
Lower jaw length	0.64	-0.19	2.36	-0.33
Mouth width	0.80	-0.50	-4.36	-1.40
Eye diameter	0.66	-0.61	5.74	3.13
Interorbital distance	0.78	0.48	-5.59	-0.07
Predorsal length	0.97	0.12	0.90	-7.80
Dorsal-fin base length	0.95	-0.11	7.66	-2.70
Dorsal-fin length	0.93	-0.13	-1.88	-3.96
Pectoral-fin length	0.96	0.02	-10.35	-0.52
Pectoral-fin base length	0.91	0.05	4.64	0.35
Prepectoral length	0.96	0.17	-7.18	5.32
Pelvic-fin length	0.91	0.05	9.18	2.90
Pelvic-fin base length	0.87	-0.19	1.26	-0.68
Prepelvic length	0.96	0.20	-4.10	-1.20
Anal-fin length	0.90	-0.21	2.49	2.53
Anal-fin base length	0.92	-0.19	-5.68	-0.58
Preanal length	0.97	0.19	-1.43	-0.16
Caudal peduncle length	0.83	0.26	3.97	0.70
Caudal peduncle depth	0.93	0.02	-8.64	-0.11
Maxillary barbel length	0.80	-0.02	-1.94	-0.86
Inrostral barbel length	0.81	0.09	-5.46	1.74
Outrostral barbel length	0.88	0.03	/	/
Eigenvalues	28.91	0.04	71.51	22.45
Percentage of total variance	77.92	6.17	75.80	24.20
Cumulative percentage	77.92	84.09	75.80	100.00
Positive correlation	/	/	0.99	0.98
